# Immune Therapy Targeting E6/E7 Oncogenes of Human Papillomavirus Type 6 (HPV-6) Reduces or Eliminates the Need for Surgical Intervention in the Treatment of HPV-6 Associated Recurrent Respiratory Papillomatosis

**DOI:** 10.3390/vaccines8010056

**Published:** 2020-01-29

**Authors:** Charu Aggarwal, Roger B. Cohen, Matthew P. Morrow, Kimberly A. Kraynyak, Albert J. Sylvester, Jocelyn Cheung, Kelsie Dickerson, Veronique Schulten, Dawson Knoblock, Elisabeth Gillespie, Joshua M. Bauml, Jian Yan, Malissa Diehl, Jean Boyer, Michael Dallas, J. Joseph Kim, David B. Weiner, Jeffrey M. Skolnik

**Affiliations:** 1 Division of Hematology Oncology, University of Pennsylvania, Philadelphia, PA 19104, USA; Charu.Aggarwal@pennmedicine.upenn.edu (C.A.); roger.cohen@uphs.upenn.edu (R.B.C.); joshua.bauml@uphs.upenn.edu (J.M.B.); 2 Inovio Pharmaceuticals, Inc., Plymouth Meeting, PA 19462, USA; kkraynyak@inovio.com (K.A.K.); asylvester@inovio.com (A.J.S.); jcheung@inovio.com (J.C.); Kelsie.Dickerson@inovio.com (K.D.); Veronique.Schulten@inovio.com (V.S.); dawsondmg@gmail.com (D.K.); Elisabeth.Gillespie@inovio.com (E.G.); jyan@inovio.com (J.Y.); mdiehl@inovio.com (M.D.); jboyer@inovio.com (J.B.); kim@inovio.com (J.J.K.); Jeffrey.Skolnik@inovio.com (J.M.S.); 3 The Wistar Institute Vaccine and Immunotherapy Center, Philadelphia, PA 19104, USA; dweiner@wistar.org

**Keywords:** HPV, immunotherapy, RRP

## Abstract

Background: Recurrent respiratory papillomatosis (RRP) is a rare disorder characterized by the generation of papillomas of the aerodigestive tract, usually associated with human papilloma virus (HPV) subtypes 6, 11. INO-3106 is a DNA plasmid-based immunotherapy targeting E6 and E7 proteins of HPV6, in order to create a robust immune T cell response. Methods: Testing of INO-3016 in animal models confirmed immunogenicity of the DNA-based therapy. A single-site open-label Phase 1 study was initiated for patients with HPV6-positive RRP. Patients were dosed with INO-3106 with or without INO-9012, a DNA plasmid immunotherapy that encodes IL-12, delivered intramuscularly (IM) in combination with electroporation (EP) with the CELLECTRA^®^ device. Patients received an escalating dose of INO-3106, 3 mg once and then 6 mg for three additional doses, each dose three weeks apart, with the third and fourth doses co-administered with INO-9012. The primary objective of the study was to evaluate the safety and tolerability of INO-3106 with and without INO-9012. The secondary objective was to determine cellular immune responses to INO-3106 with and without INO-9012. Exploratory objectives included preliminary clinical efficacy to the therapy. Results: Three patients were enrolled in this study, of which two had RRP. Study therapy was well-tolerated, with no related serious adverse events and all related adverse events (AEs) were low-grade. Injection site pain was the most common related AE reported. Immunogenicity was evidenced by multiple immune assays showing engagement and expansion of an HPV6-specific cellular response, including cytotoxic T cells. Preliminary efficacy was demonstrated in patients with RRP in the form of reduction in need for surgical intervention for papilloma growth. Prior to intervention, both patients required surgical intervention approximately every 180 days. One patient demonstrated a greater than three-fold increase in surgery avoidance (584 days) and the other patient remains completely surgery-free as of the last contact at 915 days, a greater than 5-fold increase in surgery interval. Conclusion: INO-3106 with and without INO-9012 was well tolerated, immunogenic and demonstrated preliminary efficacy in patients with HPV6-associated RRP aerodigestive lesions. Further clinical study is indicated.

## 1. Introduction

Human papilloma virus associated (HPV+) malignancies are an emerging global epidemic [1]. HPV associated aerodigestive precancerous lesions and malignancies may occur in the oropharynx, larynx, and upper respiratory tract. While the role of HPV6 in the etiology of a majority of aerodigestive malignancies remains unclear, its role is widely accepted as being causally implicated in recurrent respiratory papillomatosis (RRP) [2,3,4], the most common benign tumor of the laryngeal epithelium. RRP is rare, with an incidence rate estimated at 1.8 per 100,000 adults in the United States [5]. Although most lesions are benign, some undergo malignant transformation, and patients with RRP have a higher risk of developing laryngeal neoplasias and carcinomas [6].

The clinical course of RRP can vary widely amongst affected individuals. Selection of treatment, including active monitoring without treatment, surgery, radiation therapy, or a combination, depends on a number of factors. Usually, repeated surgical removal of papillomas for symptomatic management remains the mainstay of treatment [7]. In a few cases, malignant transformation may occur, which is usually associated with a dismal prognosis. A few such patients with malignant disease may be candidates for salvage therapies, including potentially definitive surgery [8]. Selected patients in this setting may benefit from radiation although the morbidity of this approach is substantial [9].

Current treatment of HPV6-related RRP and invasive malignant diseases could potentially be improved with the addition of HPV-specific immunotherapy. Available preventive HPV vaccines can generate neutralizing antibodies against the HPV major capsid protein L1, but they have not demonstrated therapeutic effects on HPV infection or existing lesions and are unlikely to engender a cytolytic T-cell response [10]. HPV-specific immunotherapy, on the other hand, may have therapeutic potential to eliminate preexisting lesions and infections by generating immunity against the HPV virus itself and HPV infected cells. HPV E6 and E7 oncoproteins represent ideal targets for this type of therapeutic intervention because of their constitutive expression in HPV associated tumors and their crucial role in the induction and maintenance of HPV associated diseases [11]. To that end, our previous clinical studies of HPV-specific immunotherapy engendering T cell responses have shown to translate in clinical benefit for woman exhibiting advanced dysplastic cervical lesions as well as patients with HPV associated squamous cell carcinoma of the head and neck [11,12].

**Figure 1:**
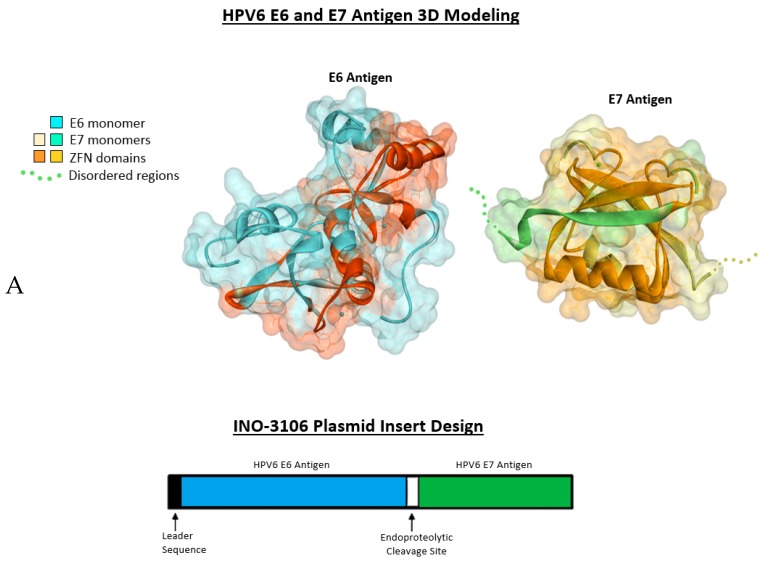

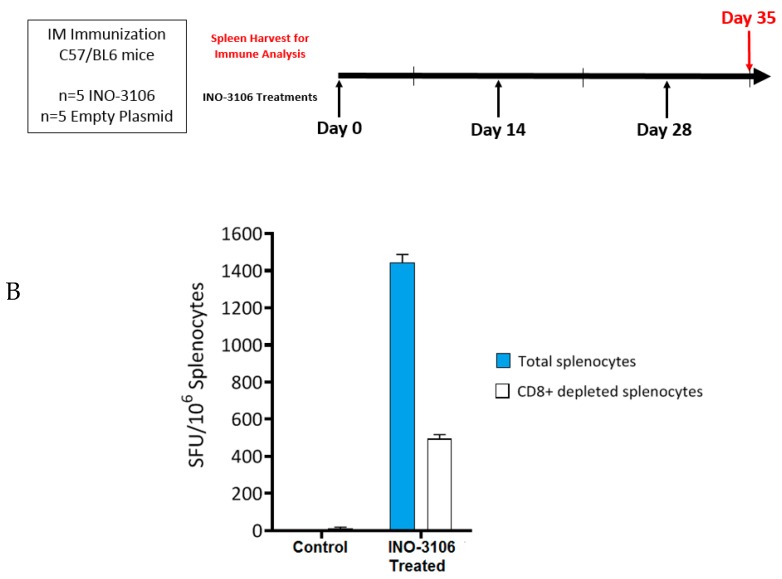
(**A**) 3D models of the HPV6 E6 and HPV6 E7 SynCon antigens with SynCon Insert. Upper panel: E6 is modeled as a monomer and the ordered C-terminal region of E7 is modeled as a homodimer. The disordered N-terminal is indicated in the figure. Both are visualized in ribbon format with side chains and a transparent solvent-accessible surface. Zinc finger motifs on both models are annotated. Lower panel: a simplified diagram of the INO-3106 transgene encoding for the HPV6 Syncon E6 and E7 antigens. A cleavage site is included between the antigens to ensure separation and individual shuttling to the immunoproteosome. (**B**) CD8^+^ mediated cellular immune response induced by INO-3106 in C57BL/6 mice. Upper panel – immunization schedule of the mouse study of INO-3106. Lower panel: Frequencies of HPV6 E6 and E7-specific IFN-γ spot forming units (SFU) per million total or CD8 depleted splenocytes were determined by ELISpot assay.

In this study, we evaluated INO-3106, a novel HPV6-specific immunotherapy consisting of synthetic consensus DNA sequences encoding for HPV6 E6 and E7 (Figure 1), proteins necessary for HPV6 induced transformation of cancers and tumor maintenance. Synthetic DNA plasmids offer several potential advantages as an immunotherapy platform, including the ability to elicit potent immune responses without evidence of genome integration, a favorable safety profile, stability and relative ease in manufacturing [13]. HPV16/18-specific therapy (VGX-3100, Inovio Pharmaceuticals, Inc.) designed and evaluated based on the same synthetic consensus platform, has demonstrated cellular immune responses that correlated with clinical benefit in the form of dysplastic lesion regression and elimination of HPV16/18 infection in a large, double-blind, randomized placebo-controlled Phase 2b study and now support Phase 3 clinical trials targeting HPV16 and 18 associated diseases [14].

Preclinical studies have shown that the immunogenicity of DNA vaccines can be substantially increased by the use of cytokine adjuvants [15,16,17,18,19]. Importantly, an engineered plasmid IL-12 genetic adjuvant has been shown to enhance immunogenicity in humans when delivered using the CELLECTRA^®^ device [20,21]. We have established in multiple clinical studies targeting both skin delivery as well as local muscle that delivery of optimized DNA via the CELLECTRA^®^ device is a highly reproducible method of generating immunity in human beings in a rapid fashion for various purposes ranging from induction prophylactic settings as well as therapeutic approaches [20,21,22,23,24,25,26,27,28].

Here, we present data suggesting that plasmid-encoded HPV6 E6 and E7 antigens are immunogenic in mice when delivered intramuscularly using the CELLECTRA^®^ device and that this immune response is primarily driven by CD8+ T cells. These data supported the initiation of a clinical trial of this plasmid (INO-3106) and we additionally report on the safety and immunogenicity of a pilot study of INO-3106 with or without INO-9012 (IL-12 gene adjuvant) delivered intramuscularly (IM) via EP with the CELLECTRA^®^ device in patients with HPV6 associated RRP. The data from this study suggest that immunotherapy with INO-3106 and IL-12 gene adjuvant could be a non-invasive immune mediated treatment option for patients with RRP and further clinical studies are warranted. 

## 2. Materials and Methods

### 2.1. Antigenic 3D Modeling

Comparative models were constructed using Bioluminate (Release 2019-2, Schrödinger, New York, NY, USA) and visualized with Discovery Studio Visualizer (Dassault Systèmes BIOVIA, San Diego, CA, USA).

### 2.2. Immunization of Mice

Female C57/BL6 mice between 6 and 8 weeks old were purchased from The Jackson Laboratory. Their care was in accordance with the guidelines of the National Institutes of Health and the University of Pennsylvania Institutional Care and Use Committee (IACUC). Mice (*n* = 5) were immunized with the HPV6 E6/E7 plasmid (pGX-3003) and the empty vector group served as negative control. Each mouse received three doses of 20µg of each plasmid at 14 day intervals. Animals were sacrificed one week after the final immunization for immune analysis. The DNA constructs were delivered intramuscularly using the CELLECTRA^®^ constant current electroporation device (Inovio Pharmaceuticals). 

### 2.3. IFN-γ ELISpot Assay

Mice were sacrificed one week after the third immunization and the splenocytes were collected. The standard IFN-γ ELISpot assay was performed as previously described [29]. A set of peptides spanning the HPV6 E6 and E7 antigens, each containing 15 amino acid residues overlapping with 8 amino acids, were synthesized from GenScript. Each set of peptides was pooled at a concentration of 2 µg/mL/peptide into two pools as antigens for specific stimulation of the IFN-γ release. The average number of spot forming cells (SFC) was adjusted to 1 × 10^6^ splenocytes. 

### 2.4. CD8+ T-Cell Depletion of Mouse Samples

CD8 lymphocytes were depleted from splenocytes by using immune-magnetic beads coated with antibody to CD8 (Dynal Biotech Inc., Lake Success, NY, USA) following manufacturer’s instructions. After depletion of CD8+ T-cells, IFN-γ ELISpot assay was performed as described above.

## 3. Clinical Study

### 3.1. Clinical Study Population

This was a prospective, open-label, Phase 1 study conducted at the Abramson Cancer Center at the University of Pennsylvania. Male and female patients at least 18 years old were considered for enrollment. To be eligible, patients must have histologically documented HPV6-associated aerodigestive papilloma, premalignant lesion or have aerodigestive invasive malignancy with most recent therapy (e.g., radiation, chemotherapy) completed at least two months prior to first dose of study treatment. Patients must have ECOG 0-1, with adequate liver, renal, hepatic and bone marrow function. Patients were excluded if there was evidence of immunosuppression or anticipated use of immunosuppressive agents, required use of systemic steroids, presence of cardiac pre-excitation syndromes, or were pregnant or breast-feeding. Written informed consent was obtained from each patient prior to performing any assessments. The clinical trial was conducted according to the ethical guidelines of the Declaration of Helsinki and was approved by the University of Pennsylvania IRB.

### 3.2. Immunotherapy and Electroporation Using CELLECTRA^®^ Device

INO-3106 is a DNA plasmid encoding for the E6 and E7 proteins of HPV type 6, formulated in sterile water for injection. INO-9012 consists of a DNA plasmid encoding for synthetic human IL-12 (p35 and p40 subunits) also formulated in sterile water for injection. Both INO-3106 and INO-9012 were designed using proprietary technology (Inovio Pharmaceuticals, Inc.) as described previously [27,28]. The CELLECTRA^®^ 2000 adaptive constant current electroporation device (Inovio Pharmaceuticals, Inc.) delivers three 52 millisecond controlled electric pulses, spaced in 1 s intervals, through a sterile, disposable array to the injection site. When inserted into tissue, the needle array centers around the site of immunotherapy injection and creates transient pores within the cell membrane to enhance cell transfection. INO-3106 with or without INO-9012 was delivered intramuscularly in a 1 mL volume followed immediately by EP with the CELLECTRA^®^ device. Treatment or dose is defined as injection of DNA plasmids followed by EP.

### 3.3. Study Design

Following informed consent, each patient was assigned a unique patient identification code. Screening procedures to determine eligibility and collect baseline characteristics were completed within 28 days prior to first dose. Patients received escalating doses of INO-3106, of which the first dose (Day 0) delivered 3 mg of INO-3106, the second dose (Week 3) delivered 6 mg of INO-3106, and the third (Week 6) and fourth (Week 9) doses delivered 6 mg of INO-3106 with 1 mg of INO-9012. Each dose was delivered three weeks apart to allow for observation of development of any grade 2 or higher related systemic adverse events (AEs). In total, participation for all patients included a 9-week treatment period followed by a 6 month long term follow-up period from the last dose.

The primary objective of the study was to evaluate the safety and tolerability of INO-3106 with and without INO-9012. The secondary objective was to determine the humoral and cellular immune responses to INO-3106 with and without INO-9012, and the exploratory objective was to assess preliminary clinical efficacy to the treatment, as well as to associate efficacy with immune cell infiltration in post-dose tissue, if possible. 

The study was registered on ClinicalTrials.gov with the identifier NCT02241369. The study protocol conforms to the ethical guidelines of the 1975 Declaration of Helsinki and was reviewed and approved by the center’s Institutional Review Board.

### 3.4. Safety Assessments

Local and systemic adverse events (AEs), vital signs, 12 lead electrocardiograms (ECGs) and the development of laboratory abnormalities were monitored from the date of informed consent through the last follow-up visit. In particular, injection site reactions, including pain, itching, erythema, induration and bruising were assessed on the day of each treatment and for 7 consecutive days post-treatment. Patients were queried at each visit regarding the occurrence of new AEs or disease and use of concomitant medications. All events were graded in accordance with the Common Terminology Criteria for Adverse Events (CTCAE), version 4.03 and coded with MedDRA version 21. Laboratory parameters including hematology, coagulation, serum chemistry (including liver function) and creatine phosphokinase (CPK), were monitored throughout the study and assessed locally at the center. 

Further enrollment and treatment was to be halted if one third or more patients experienced a related event requiring expedited reporting: any patient experienced a serious adverse event (SAE), unexpected grade 4 toxicity, potentially life-threatening AE or death assessed as related to study treatment; three or more patients experienced the same related grade 3 or 4 AE; or if any patient reported a grade 3 anaphylaxis.

### 3.5. HPV6 Specific ELISA

A standardized binding ELISA was performed to measure IgG antibodies from patient’s sera against HPV6 E7 protein. Briefly, plates were coated with 1 ug/mL HPV6 E7 in PBS overnight at 4 °C. The following day, plates were washed and blocked with 3% BSA. After 2h incubation at room temperature, plates were washed again and patient serum samples were added in a 3-fold dilution series from 1:25 to 1:18,225. Each dilution was tested in triplicate. Samples were incubated for 2h at room temperature, followed by a washing step and 1h incubation with detection antibody goat anti-human IgG-HRP at 1:5000. Finally, plates were washed and developed using TMB substrate followed by TMB stop solution. Optical densities (OD) were read on a kinetic microplate reader. Antibody titers were determined and positivity was considered if the average OD of a sample post vaccination was greater than the average OD at baseline plus 2.5 times SD of OD at baseline at the corresponding dilution. Number “1” instead of “0” is used to indicate negative results.

### 3.6. HPV6 Specific Flow Cytometry

PBMCs were recovered after cryopreservation overnight in cell culture medium and spun, washed and re-suspended the following day. After counting, 1 × 10^6^ PBMCs were plated into a 96-well plate in R10 medium from patients with sufficient sample. For antigen specific responses, cells were stimulated 5 days with a combination of peptides corresponding to HPV6 E6 and E7 that had been pooled at a concentration of 2 µg/mL, while an irrelevant peptide was used as a negative control (OVA) and concanavalin A was used as a positive control (Sigma-Aldrich). No co-stimulatory antibodies or cytokines were added to cell cultures at any point. At the end of the 5 day incubation period, cells were stained for CD3-BUV737, CD4-APC-Cy7, CD14-BUV395, CD-16-BUV395, CD137-APC, Granulysin-AF488 CD-19-BUV395, CD38-BV786, CD8-BV650, granzyme B-AF700 (BD Biosciences), Granzyme A-PECy7 (ThermoFisher), PD-1-PEDazzle, perforin-BV421, Ki67-BV605 and CD69-BV711 (BioLegend). Staining for extracellular markers (CD4, CD8, CD137, CD69, CD38, PD-1) occurred first, followed by permeabilization to stain for the remaining markers. CD3 was stained intracellularly to account for downregulation of the marker following cellular activation. Acquired data were analyzed using the FlowJo software version X.0.7 or later (Tree Star).

### 3.7. HPV6 Specific PBMC Stimulation for Gene Expression Analysis

For short term stimulation, Cryopreserved PBMCs were thawed, rested overnight, and stimulated for 22 h at 37 °C, 5% CO_2_ and 95% humidity with either DMSO (negative control) or HPV6 E6 and E7 overlapping peptide pools (OLPs). Following stimulation, culture supernatants were collected and stored at −20 °C. Cells were then lysed using Buffer RLT (Qiagen) and stored at −80 °C.

For long term stimulation, Cryopreserved PBMCs were thawed, rested overnight, and stimulated at 37 °C, 5% CO_2_ and 95% humidity for 11 days with HPV6 E6 and E7 OLPs. On days 1, 4, 6 and 8, fresh media containing IL-2 and IL-7 was added at 10 U/mL and 10 ng/mL, respectively. On day 11, PBMCs were washed and rested overnight at 37 °C, 5% CO_2_ and 95% humidity. Following overnight rest, PBMCs were re-stimulated with HPV6 E6 and E7 OLPs for 22 h with either DMSO (negative control) or HPV6 E6 and E7 OLPs. At the end of the 22 h stimulation, cell supernatants were collected and stored at −20 °C. Cells were then lysed and stored at −80 °C.

### 3.8. Multiplexed Gene Expression Analysis

Cell lysates were thawed in batches of 12 as per manufacturer instructions and hybridized to capture probes and fluorophore-barcoded reporter probes using the nCounter (NanoString) GX Human Immunology V2 panel, which consists of 594 genes plus 15 internal reference controls. Samples were then placed in the automated nCounter Prep Station (Nanostring) for hybridization of capture probes to a translucent cartridge, after which gene expression was measured by the nCounter Digital Analyzer (Nanostring) via direct counts of reporter probes in each sample lane.

### 3.9. Statistical Methods

Regarding the CD8+ mediated cellular immune response induced by INO-3106 in C57BL/6 mice, the frequencies of HPV6 E6 and E7-specific IFN-γ spot forming units (SFU) per million determined by ELISpot assay total splenocytes were compared to CD8 depleted splenocytes using the Wilcoxon signed-rank test.

## 4. Results 

### 4.1. INO-3106 Displays Immunogenicity in Animal Models

In order to determine if a plasmid encoding the HPV6 E6 and E7 antigens (Figure 1) exhibited immunity-inducing potential, we employed the C57BL/6 mouse line as a model of active cellular immune response. Accordingly, mice were immunized with 20 µg of INO-3106, three times two weeks apart intramuscularly via the CELLECTRA^®^ device (Figure 1A). Mice were then assessed for HPV6 E6 and E7 specific T cell activity using the IFN-γ ELISpot assay. Immunization with INO-3016 indeed induced strong cellular immune response (1443+44 SFU/10^6^ splenocytes, Figure 1B). As the induction of a cellular response to the E6 and E7 antigens that is capable of eliminating virally infected cells is likely to necessitate a CD8+ T cell component, we examined the section of the T cell compartment in our mouse model. CD8 depletion of mouse splenocytes confirmed that CD8+ T cells were indeed responsible for the bulk of IFN-γ secretion detected in mice receiving INO-3016 via electroporation. This was evidenced by the fact that the number of SFU/10^6^ splenocytes was reduced to 496 +21 after CD8+ depletion, indicating that the cellular immune response induced by the INO-3106 is mediated mainly by CD8+ T-cells (Figure 1B, p = ns).

### 4.2. INO-3106 Is Tolerable, Immunogenic and Potentially Effective in Patients with RRP

#### 4.2.1. Patient Characteristics and Disposition

Confirmation of the ability of INO-3106 to induce immune responses in animal models generated sufficient evidence of activity to support the initiation of a clinical trial for HPV6-associated recurrent respiratory papillomatosis and associated malignancies. In total, four patients were consented and screened for eligibility for the trial. Three patients met all inclusion and exclusion criteria and were enrolled from October 2014 to September 2017. Of those three, two patients presented with HPV6-associated RRP (both with disease in the vocal cords) and one patient had an invasive malignancy (data not presented in this manuscript). Both RRP patients completed all four doses, receiving 3 mg of INO-3106 on Day 0, 6 mg of INO-3106 at Week 3, and 6 mg of INO-3106 with 1 mg of INO-9012 at Weeks 6 and 9, all delivered intramuscularly via the CELLECTRA^®^ device. Both patients completed the 6 month long term follow-up period following their last dose of treatment. 

#### 4.2.2. Safety and Tolerability of INO-3106 and INO-9012 with Electroporation (EP)

INO-3106 and INO-9012 delivered via EP was well-tolerated. Treatment-emergent AEs included injection site pain (three related grade 1 events), pyrexia (one unrelated grade 1 event) and urinary tract infection (one unrelated grade 2 event). All patients reported injection site pain, in most cases treated with medication leading to resolution. One treatment-emergent SAE of grade 3 monoplegia requiring hospitalization was reported on the study but was assessed to be unrelated to study treatment. No patients withdrew from receiving continued study treatment or from continued participation in the study due to an AE nor due to intolerability of EP. No grade 4 events nor deaths were reported during the course of the study. All patients experienced changes in laboratory parameters, the majority of which included slight fluctuations in hematology values, but all abnormal laboratory values were determined to be not clinically significant.

### 4.3. INO-3106 Induces Humoral Immune Reactivity in Patients with RRP 

**Figure 2:**
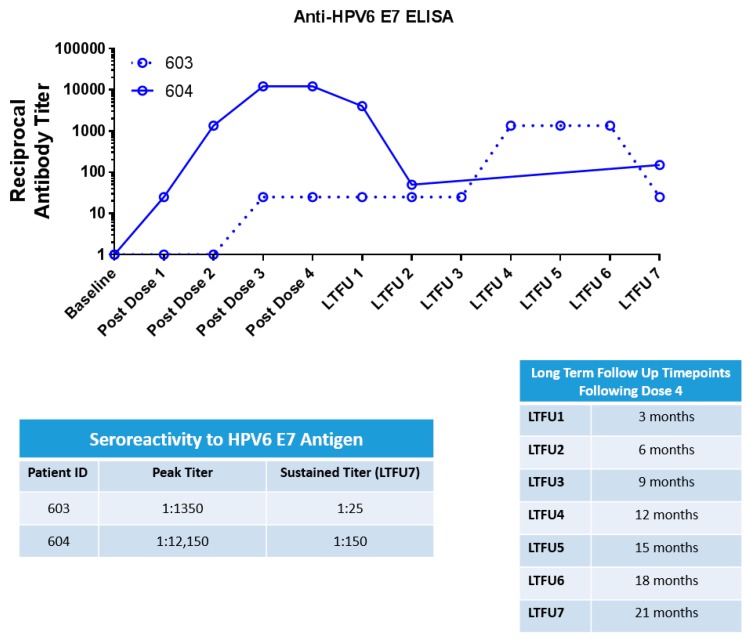
INO-3106 drives humoral responses in recurrent respiratory papillomatosis (RRP) patients. Upper panel: longitudinal assessment of antibodies against HPV6 E7 antigen are shown for patient 603 (dotted line) and 604 (solid line). Lower panel: peak (highest recorded value) and sustained (final value above study entry) titers for each patient are shown.

While the E6 and E7 antigens do not constitute direct targets for antibody binding on infected cells due to their internal (as opposed to cell surface) localization, the measure of the induction of a humoral response to antigens encoded by INO-3106 serves as an indicator of treatment-based changes in the immune system of enrolled patients. To that end, sera from treated patients were isolated and used in a standard binding ELISA to determine if treatment with INO-3106 was impacting antibody responses to HPV6 antigens. Results presented in Figure 2 show that both RRP patients showed elevations in seroreactivity against the HPV6 E7 antigen as evidenced by increases in reciprocal endpoint antibody titers. Both patients showed robust peak antibody titers reaching the thousands (1:1350 for patient 603, 1:12,150 for patient 604) and both showed sustained presence of these HPV6 specific antibodies above baseline through the last recorded timepoint, 21 months following completion of INO-3106 dosing.

### 4.4. INO-3106 Induces the Expression of Activation Markers and Lytic Proteins in T Cells from Treated Patients with RRP 

**Figure 3:**
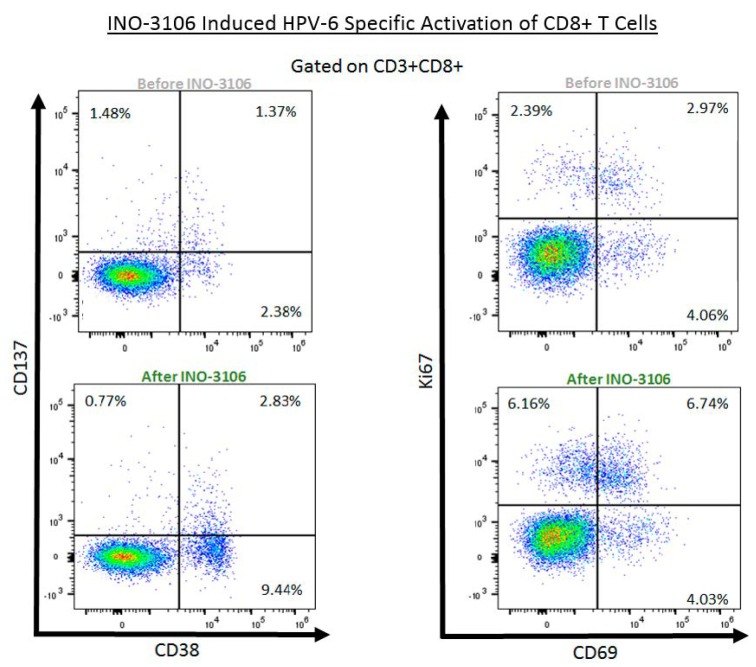
INO-3106 Activates HPV6-specific cytotoxic lymphocytes cells in RRP patients. Flow cytometry was performed to assess activation marker expression on HPV6-specific CD8+ T cells taken from patients before and after immunotherapy. Expression of CD137 and CD38 before (upper panel) and after (lower panel) treatment with INO-3106 in patient 604 are noted in the left column. Expression of Ki67 and CD69 before (upper panel) and after (lower panel) treatment with INO-3106 in patient 604 are noted in the right column.

It is understood that a cytolytic response by CD8+ T cells is a key component of an immune response that will control and eliminate virally infected cells. We therefore performed flow cytometry on PBMCs from patients 603 and 604 with sufficient sample isolated prior to and after dosing with INO-3106 to assess the ability of HPV6 specific CD8+ T cells to load granzymes and perforin in response to treatment. To that end, we analyzed the CD8+ T cell compartment of both patients for immune activation via antigen-specific expression of cell surface markers such as CD38, CD69, CD137 and Ki67 (Figure 3) as well as for lytic potential as determined by the presence of granulysin (Gnly), granzyme A (GrzA), granzyme B (GrzB) and perforin (Prf) after in vitro stimulation with cognate antigens. Table 1 and Table 2 show antigen specific regulation of these markers prior to and following treatment with INO-3106. Patient 603 exhibited robust elevations of a variety of CD8+ T cells expressing activation markers concomitant with lytic proteins. Most notably, expression of CD38 and/or Ki67 in combination with markers of lytic potential such as granzyme A, granzyme B and perforin increases dramatically after treatment with INO-3106, reaching values surpassing 3% of total CD8+ T cells being specific to HPV6 E6 and E7 antigens (Table 1, Figure 3). Conversely, patient 604 (Table 2, Figure 3) showed smaller elevations in CD8+ T cell responses from a magnitude perspective when compared to patient 603. Interestingly, while smaller in magnitude than patient 603, the phenotypes of the putative CTLs induced in patient 604 suggest the possibility of a more highly active CD8+ T cell, as the populations that were most likely to be increased after treatment consisted of three (CD38, CD137, Ki67) activation markers or concomitant expression of all four (CD69 in addition to the previous three). Co-expression of this number of activation markers concomitantly is far more rare in our experience [29,30,31,32,33,34] and previous interrogation of CD8+ T cells expressing multiple activation markers has suggested these cells express granzymes and can effectively induce apoptosis in targets expressing cognate antigen [35]. Indeed, this last idea is further supported when one notes that patient 604 showed increases in expression not just in granzymes and perforin, but in granulysin as well. While the limitations of this small sample size need to be taken into context, the results of this assessment suggest that INO-3106 potentially led to the induction of CD8+ T cells capable of activation in the context of antigenic exposure and that these cells were capable of granzyme, perforin and granulysin synthesis, thus exhibiting a clear HPV6 specific CTL phenotype. 

### 4.5. INO-3106 Changes Immune Transcriptional Profiles of T Cells in RRP Patients 

**Figure 4:**
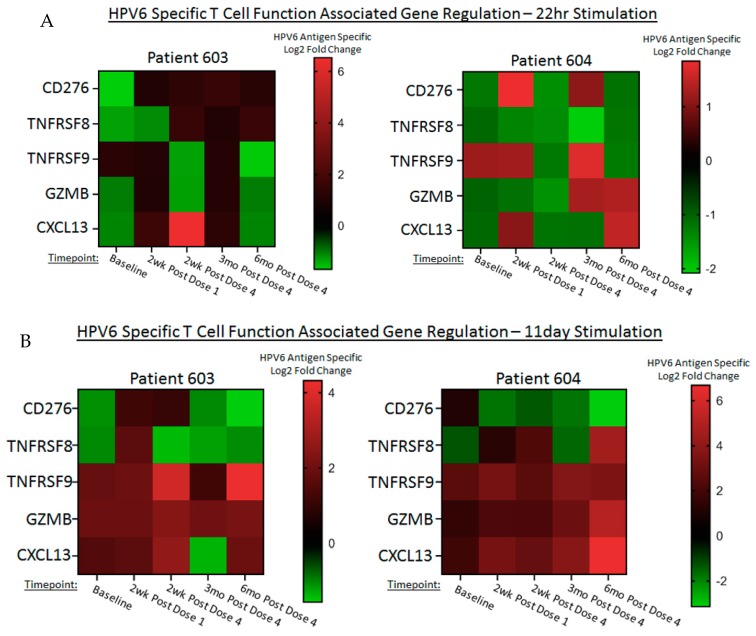
Immune gene transcripts are differentially regulated in an HPV6 specific fashion after treatment with INO-3106. Heat maps showing fold difference of differentially expressed genes in stimulated versus unstimulated cells pre and post vaccination. (**A**) Fold change in gene expression (≥ 2-fold) in cells stimulated with peptide pool versus medium alone for 24 h. (**B**) Fold change in gene expression (≥ 2-fold) after 11 days of T cell expansion followed by restimulation of cells with peptide pool versus medium alone for 24 h. Data are transformed to log_2_ fold change, with red indicating upregulation and green indicating downregulation.

As an additional mechanism of profile HPV6 specific immune responses, we performed short-term stimulations of patient PBMCs (24 h), followed by an analysis of immune gene transcripts that were found to be specifically regulated in response to stimulation with HPV6 E6 and E7-derived peptide pools. For patients 603 and 604, gene transcription was mostly associated with upregulation of a pro-inflammatory signature post immunotherapy that included key factors surrounding T cell activity, as well as a chemoattractant for B cells (Figure 4A). Specifically, expression of CD276, a protein with an established roll in T cell suppression was down regulated or unchanged for most timepoints for both patients. Additionally, HPV6 specific downregulation of the pro-apoptotic receptor TNFRSF8 (also known as CD30) was noted for both patients. Conversely, markers indicative of T cell activation (TNFRSF9/CD137) and potential lytic activity (Granzyme B) were noted as being stable or elevated relative to baseline for both patients. This combination of immune gene regulation therefor constitutes a shift away from suppression and towards augmentation of T cell activity. Likewise, the chemokine CXCL13 which plays a role in B cell migration and activity was also noted as frequently stable or elevated relative to baseline, which keeps with the antibody data presented earlier suggesting that INO-3106 activates the humoral arm of patients’ immune systems as well.

*In vitro* culture for 11 days allowed for a look at antigen- specific immune responses in the face of prolonged antigen stimulation – a format that more closely mimics what immune cells infiltrating into RRP tissues would be exposed to. The construction of these assay stimulation conditions favor T cell expansion above all other cell types including B cells and other APCs. Overall, both patients showed frequent downregulation of CD276, consistent with a reduction in inhibitory factors for immune activation. TNFRSF8/CD30 showed varying degrees of transcriptional change, with patient 603 exhibiting more consistent down regulation. Importantly, both patients showed strong and sustained upregulation in the factors associated with T cell activation, lytic potential and B cell homing and activity: TNFRSF9/CD137, Granzyme B and CXCL13 (Figure 4B). Thus even in the face of prolonged antigenic stimulation, the immune systems of RRP patients treated with INO-3106 retain the ability to exhibit proinflammatory gene signatures, including markers of activation and lytic potential.

### 4.6. INO-3106 Reduces the Need for Surgical Intervention for the Treatment of RRP

**Figure 5:**
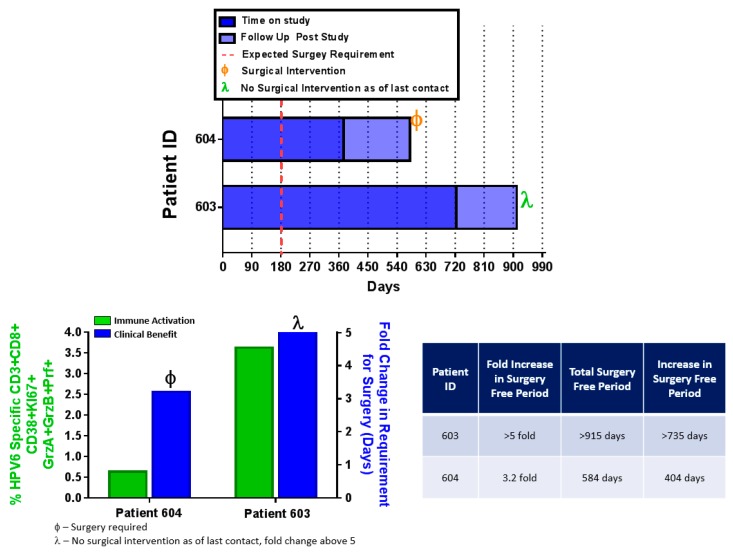
Treatment of RRP patients with INO-3106 imparts clinical benefit in the form of avoidance of surgery. Top panel: Swimmers plot indicating the length of time in Days that patients 604 and 603 were surgery-free. The dotted red line indicates the timepoint surgery would be expected based on previous surgery frequencies prior to intervention with INO-3106, indicates the timepoint at which patient 604 required surgery, and indicates that as of the indicated timepoint, patient 603 remains surgery free. Bottom left panel: Green bars track to the left y-axis and indicate the magnitude of HPV6-specific CD8+ T cells expressing CD38, Ki67, Granzyme A, Granzyme B and Perforin. Blue bars track to the right y-axis and indicate the fold-change in surgery-free time relative to the expected surgery frequencies for these patients. Bottom right – the chart indicates patient ID, fold increase in surgery free time, total surgery free time, and the increase in surgery free time experienced by these patients after treatment with INO-3106.

Prior to entry into the study, patients 603 and 604 required surgical frequent intervention to remove respiratory papillomas at an estimated mean of every 180 days, with true variance being difficult to accurately determine, as the timing of surgery was dictated by emergence of symptoms. Assuming recurrence were to continue in this fashion, the expected number of required surgical interventions over the course of the study would be four for patient 603 and two for patient 604. However, over the entirety of the study, neither patient required surgical intervention for the removal of airway papillomas, constituting a clinical change in the need for intervention in the treatment of this disease. Post-study follow-up of these patients reveals that patient 603 has not required surgical intervention in the treatment of disease at the time of this publication, totaling more than 915 days without surgery. After 584 days, patient 604 had a recurrence of disease that did require surgical intervention to appropriately treat, and an overall reduction in surgery frequency great than 3-fold (Figure 5). The difference in the outcomes of these patients prompted us to examine if any of the immunology data generated while on study would have suggested a differential clinical response to treatment. Assessment of flow cytometry indicated that patient 604 had more robust immune activity in the form of HPV6-specific CTLs than patient 603 (Figure 5). It is therefore possible that the difference in both the magnitude of response and pattern of activation marker expression on patient CTLs could associated with the durability of clinical impact. Further studies of an immunotherapy based intervention for RRP will be able to more appropriately address this concept.

## 5. Discussion 

Here, we report that a plasmid encoding HPV6 E6 and E7 antigens (INO-3106) delivered by the CELLECTRA^®^ device was immunogenic in preclinical models, allowing for the support of a clinical trial for HPV6 associated recurrent respiratory papillomatosis. The resulting Phase 1 clinical trial of INO-3016 with and without IL-12 DNA adjuvant administered intramuscularly and delivered via electroporation by the CELLECTRA^®^ device treated two patients with HPV6 associated recurrent respiratory papillomatosis. Administration of the immunotherapy was well-tolerated. There were no treatment-related SAEs and the most frequent treatment-emergent AEs were injection site reactions. All patients showed induction of cellular responses to the HPV6 E6 and E7 antigens as demonstrated by at least one immunological assessment. Notably, both evaluable RRP patients derived clinical benefit from treatment with INO-3106, mainly in the form of delayed treatment intervention (e.g., surgery) relative to their pre-study surgery frequencies. Moreover, the fact that the patient who exhibited more robust cellular activity after INO-3106 treatment remains surgery free while the patient with less robust cellular activity delayed but did not completely avoid surgery suggests a possible causal relationship between the induction of an HPV6-specific cellular response and the type/duration of clinical benefit. With the understanding that the evaluation of two patients does not sufficiently power a study to reject a null hypothesis, these results are nonetheless encouraging and present the idea that additional dosing to continue to boost the cellular response may be preferable in this treatment setting. Such a hypothesis requires further testing in order to validate, however. 

Treatment with INO-3106 resulted in the induction of HPV6-specific cellular responses across a variety of immunoassays. The confirmation of production of IFN-γ using ELISpot as well as the confirmation of expression of activation markers concomitant with synthesis of granzyme and perforin on CD8+ T cells via flow cytometry suggests that INO-3106 drove the induction of a proinflammatory immune response that included T cells with hallmarks of highly activated cytotoxic lymphocytes. These results are further underscored by the observation of dynamic regulation of pro-inflammatory as well as regulatory gene transcripts in PBMCs after completion of treatment. Specifically, we observed increased expression of Granzyme B and TNFRSF9/CD137 transcripts, confirming the activity of cytotoxic lymphocytes on the transcriptomic level. The necessity for a T cell response of this nature in combating HPV-driven disease has been exemplified in two of our previous clinical trials for DNA-based immunotherapy, both of which were delivered using the CELLECTRA^®^ device. In the context of HPV-associated cervical dysplasia in a large, double-blind, placebo-controlled Phase 2b trial, clinical response to treatment with VGX-3100 (DNA immunotherapy for targeting E6/E7 of HPV16/18) in the form of regression of lesions concomitant with elimination of HPV infection was statistically associated with the presence of a robust cellular response that included IFN-γ and CD8+ T cells exhibiting phenotypic markers of cytoxicity [25]. Additionally, in another trial investigating treatment of HPV-associated squamous cell cancer of the oropharynx, a patient with metastatic cancer who achieved a complete response to treatment with nivolumab after treatment with MEDI0457 (VGX-3100 + INO-9012) was noted as having a therapy-driven robust expansion of PD1^+^ cytotoxic T cells [27]. Thus, the current study provides further evidence that HPV-specific immunotherapies delivered by the CELLECTRA^®^ device induce the generation of potent T cell responses that have the potential to clinically impact HPV-associated tumorigenesis.

The data collected from this study are the first to suggest that an HPV specific immunotherapy may be able to impact the clinical status of patients with HPV6 associated recurrent respiratory papillomatosis and act as an additional or alternative adjuvant therapy. These findings are complementary to data presented earlier this year, where administration of pembrolizumab was associated with a reduced need for routine surgical interventions [34]. Together, these findings offer early data to support the use of immunotherapeutic approaches in the management of these patients. The current standard of care for treatment for this disease is repeated surgical intervention, which presents a number of complications and is unlikely to completely eradicate lesion recurrence as latent virus may reside in adjacent tissue [34]. Other non-surgical adjuvant interventions are indicated in patients with rapid regrowth of lesions or aggressive disease, but such therapies also carry inherent risks and require further evaluation to determine optimal treatment regimens [7]. Current treatment limitations highlight the need to identify non-invasive and immune mediated approaches to treating patients with HPV positive areodigestive disease. Indeed, preventive HPV vaccines have been reported to reduce papilloma growth and extend time between interventions, however, determination of therapeutic efficacy requires continued evaluation [33]. Similarly, PD-1/PD-L1 inhibition represents a rational approach to treating RRP, but expression and impact on clinical outcome is less characterized [34].

Our study has a few important limitations that should be noted. First, the small size of the study precludes our ability to formally test a number of hypotheses. The small size of this study is likely due to the limited enrollment criteria, i.e. this was limited to patients over the age of 18 years and therefore could not capture a population of patients with Juvenile RRP, a condition that is perhaps slightly more common than in the adult population. Additionally, this was a single center study, with a small sample size, and therefore was limited in its catchment: the study was designed as a small Phase 1P study, wherein individual patients, specifically those with more severe RRP disease with inevitable continued resections and potential malignant transformation, are treated with investigational products (i.e., INO-3106 and INO-9012) otherwise not obtainable. 

## 6. Conclusions

The data generated from the present study suggest that immunotherapy with INO-3106 and IL-12 adjuvant as a non-invasive immune mediated approach may provide an option to address existing treatment deficiencies for RRP. While the current study focused on HPV6 positive disease, HPV11 is also noted as contributing to aerodigestive malignancies. Further clinical studies including a wider age range are needed to evaluate the impact of immunotherapy against both HPV types. 

## Figures and Tables

**Table 1:** CD8+ T Cell Phenotyping for Patient 603.

Markers Co-Expressed on CD8+ T Cells:	Before INO-3106 (%)	After INO-3106 (%)
CD38+Ki67+	0.49	3.95
Ki67+	0.45	3.88
CD38+Ki67+Prf+	0.53	3.93
CD38+Ki67+GrzA+Prf+	0.58	3.95
Ki67+GrzA+Prf+	0.53	3.91
Ki67+Prf+	0.48	3.84
Ki67+GrzB+Prf+	0.50	3.66
CD38+Ki67+GrzA+GrzB+Prf+	0.51	3.66
CD38+Ki67+GrzB+Prf+	0.51	3.66
Ki67+GrzA+GrzB+Prf+	0.51	3.66

Markers assessed: CD38, CD69, CD137, Ki67, GrzA, GrzB, Gnly, Prf; Key: Prf = perforin, GrzA = Granzyme A, GrzB = Granzyme B, Gnly = Granulysin.

**Table 2:** CD8+ T Cell Phenotyping for Patient 604.

Markers Co-Expressed on CD8+ T Cells:	Before INO-3106 (%)	After INO-3106 (%)
CD38+CD137+Ki67+GrzB+Prf+	0.35	0.51
CD38+CD69+CD137+ Ki67+GrzB+Prf+	0.35	0.48
CD137+Ki67+GrzB+Prf+	0.36	0.48
CD38+CD137+Ki67+ GrzA+GrzB+Prf+	0.36	0.48
CD69+CD137+Ki67+GrzB+Prf+	0.36	0.47
CD38+CD69+CD137+Ki67+ GrzA+GrzB+Prf+	0.36	0.46
CD137+Ki67+GrzA+GrzB+Prf+	0.37	0.46
CD38+CD137+GrzB+Prf+	0.41	0.49
CD38+Ki67+Gnly+GrzA+Prf+	0.00	0.08
CD38+Gnly+	0.00	0.08
